# 5-Cyclo­hexyl-2-methyl-3-phenyl­sulfinyl-1-benzofuran

**DOI:** 10.1107/S1600536811013705

**Published:** 2011-04-16

**Authors:** Hong Dae Choi, Pil Ja Seo, Byeng Wha Son, Uk Lee

**Affiliations:** a Department of Chemistry, Dongeui University, San 24 Kaya-dong Busanjin-gu, Busan 614-714, Republic of Korea; b Department of Chemistry, Pukyong National University, 599-1 Daeyeon 3-dong, Nam-gu, Busan 608-737, Republic of Korea

## Abstract

In the title compound, C_21_H_22_O_2_S, the cyclo­hexyl ring adopts a chair conformation. The phenyl ring makes a dihedral angle of 84.80 (4)° with the mean plane of the benzofuran fragment. In the crystal, mol­ecules are linked through weak inter­molecular C—H⋯O hydrogen bonds.

## Related literature

For the pharmacological activity of benzofuran compounds, see: Aslam *et al.* (2009 ▶); Galal *et al.* (2009 ▶); Khan *et al.* (2005 ▶). For natural products with benzofuran rings, see: Akgul & Anil (2003 ▶); Soekamto *et al.* (2003 ▶). For structural studies of related 3-aryl­sulfinyl-2-methyl-1-benzofuran derivatives, see: Choi *et al.* (2010 ▶, 2011 ▶).
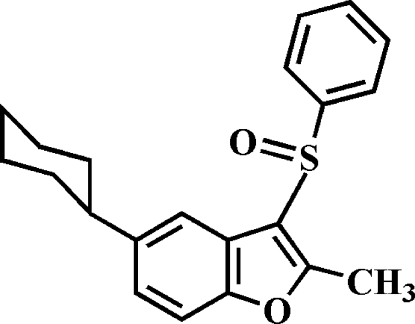


## Experimental

### 

#### Crystal data

C_21_H_22_O_2_S
*M* *_r_* = 338.45
Triclinic, 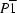

*a* = 8.8532 (2) Å
*b* = 10.2011 (2) Å
*c* = 10.4752 (3) Å
α = 90.734 (1)°
β = 111.568 (1)°
γ = 97.127 (1)°
*V* = 871.25 (4) Å^3^
*Z* = 2
Mo *K*α radiation
μ = 0.20 mm^−1^
*T* = 173 K
0.35 × 0.25 × 0.16 mm

#### Data collection

Bruker SMART APEXII CCD diffractometer
Absorption correction: multi-scan (*SADABS*; Bruker, 2009 ▶) *T*_min_ = 0.650, *T*_max_ = 0.746
15560 measured reflections
4012 independent reflections
3481 reflections with *I* > 2σ(*I*)
*R*_int_ = 0.028

#### Refinement

*R*[*F* ^2^ > 2σ(*F* ^2^)] = 0.039
*wR*(*F* ^2^) = 0.106
*S* = 1.07
4012 reflections
218 parameters
H-atom parameters constrained
Δρ_max_ = 0.44 e Å^−3^
Δρ_min_ = −0.26 e Å^−3^

### 

Data collection: *APEX2* (Bruker, 2009 ▶); cell refinement: *SAINT* (Bruker, 2009 ▶); data reduction: *SAINT*; program(s) used to solve structure: *SHELXS97* (Sheldrick, 2008 ▶); program(s) used to refine structure: *SHELXL97* (Sheldrick, 2008 ▶); molecular graphics: *ORTEP-3* (Farrugia, 1997 ▶) and *DIAMOND* (Brandenburg, 1998 ▶); software used to prepare material for publication: *SHELXL97*.

## Supplementary Material

Crystal structure: contains datablocks global, I. DOI: 10.1107/S1600536811013705/zl2365sup1.cif

Structure factors: contains datablocks I. DOI: 10.1107/S1600536811013705/zl2365Isup2.hkl

Additional supplementary materials:  crystallographic information; 3D view; checkCIF report

## Figures and Tables

**Table 1:** Hydrogen-bond geometry (Å, °)

*D*—H⋯*A*	*D*—H	H⋯*A*	*D*⋯*A*	*D*—H⋯*A*
C15—H15*B*⋯O2^i^	0.98	2.52	3.4516 (19)	159
C21—H21⋯O2^ii^	0.95	2.47	3.2793 (18)	144

Symmetry codes: (i) 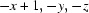; (ii) 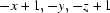.

**Fig. 1.**
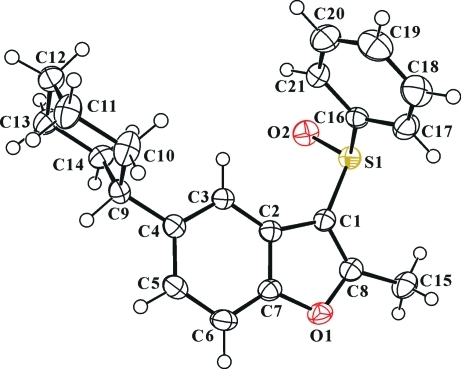
The molecular structure of the title compound with the atom numbering scheme. Displacement ellipsoids are drawn at the 50% probability level. H atoms are presented as a small spheres of arbitrary radius.

**Fig. 2.**
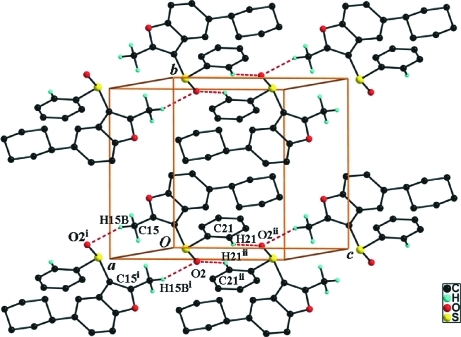
A view of the C–H···O hydrogen bonds (dotted lines) in the crystal structure of the title compound. [Symmetry codes: (i) - x + 1, - y, - z; (ii) - x + 1, - y, -z + 1.]

## supplementary crystallographic information

### Comment

Many compounds having a benzofuran ring system have attracted much attention owing to their diverse pharmacological properties such as antibacterial and antifungal, antitumor and antiviral, and antimicrobial activities (Aslam *et al.*, 2009, Galal *et al.*, 2009, Khan *et al.*, 2005). These compounds widely occur in nature (Akgul & Anil, 2003; Soekamto *et al.*, 2003). As a part of our study of the substituent effect on the solid state structures of 3-arylsulfinyl-2-methyl-1-benzofuran analogues (Choi *et al.*, 2010, 2011), we report here on the crystal structure of the title compound

In the title molecule (Fig. 1), the benzofuran unit is essentially planar, with a mean deviation of 0.004 (1) Å from the least-squares plane defined by the nine constituent atoms. The cyclohexyl ring is in the chair form. The dihedral angle formed by the mean plane of the benzofuran ring and the phenyl ring is 84.80 (4)° . The crystal packing (Fig. 2) is stabilized by weak intermolecular C—H···O hydrogen bonds; the first one between a methyl H atom and the O atom of the sulfinyl group (Table 1; C15—H15B···O2^i^), and the second one between a phenyl H atom and the O atom of the sulfinyl group (Table 1; C21—H21···O2^ii^).

### Experimental

77% 3-chloroperoxybenzoic acid (269 mg, 1.2 mmol) was added in small portions to a stirred solution of 5-cyclohexyl-2-methyl-3-phenylsulfanyl-1-benzofuran (354 mg, 1.1 mmol) in dichloromethane (30 mL) at 273 K. After being stirred at room temperature for 5h, the mixture was washed with saturated sodium bicarbonate solution and the organic layer was separated, dried over magnesium sulfate, filtered and concentrated at reduced pressure. The residue was purified by column chromatography (hexane–ethyl acetate, 2:1 v/v) to afford the title compound as a colorless solid [yield 71%, m.p. 406–407 K; *R*_f_ = 0.68 (hexane–ethyl acetate, 2:1 v/v)]. Single crystals suitable for X-ray diffraction were prepared by slow evaporation of a solution of the title compound in ethyl acetate at room temperature.

### Refinement

All H atoms were positioned geometrically and refined using a riding model, with C—H = 0.95 Å  for aryl, 1.00 Å for methine, 0.99 Å for methylene and 0.98 Å for methyl H atoms, respectively. *U*_iso_(H) = 1.2*U*_eq_(C) for aryl, methine and methylene, and 1.5*U*_eq_(C) for methyl H atoms.

### Crystal data

**Table:**

C_21_H_22_O_2_S	*Z* = 2
*M**_r_* = 338.45	*F*(000) = 360
Triclinic, *P*1	*D*_x_ = 1.290 Mg m^−^^3^
Hall symbol: -P 1	Mo *K*α radiation, λ = 0.71073 Å
*a* = 8.8532 (2) Å	Cell parameters from 6502 reflections
*b* = 10.2011 (2) Å	θ = 2.6–27.5°
*c* = 10.4752 (3) Å	µ = 0.20 mm^−^^1^
α = 90.734 (1)°	*T* = 173 K
β = 111.568 (1)°	Block, colourless
γ = 97.127 (1)°	0.35 × 0.25 × 0.16 mm
*V* = 871.25 (4) Å^3^	

### Data collection

**Table:**

Bruker SMART APEXII CCD diffractometer	4012 independent reflections
Radiation source: rotating anode	3481 reflections with *I* > 2σ(*I*)
graphite multilayer	*R*_int_ = 0.028
Detector resolution: 10.0 pixels mm^-1^	θ_max_ = 27.5°, θ_min_ = 2.0°
φ and ω scans	*h* = −11→11
Absorption correction: multi-scan (*SADABS*; Bruker, 2009)	*k* = −13→13
*T*_min_ = 0.650, *T*_max_ = 0.746	*l* = −13→13
15560 measured reflections	

### Refinement

**Table:**

Refinement on *F*^2^	Primary atom site location: structure-invariant direct methods
Least-squares matrix: full	Secondary atom site location: difference Fourier map
*R*[*F*^2^ > 2σ(*F*^2^)] = 0.039	Hydrogen site location: difference Fourier map
*wR*(*F*^2^) = 0.106	H-atom parameters constrained
*S* = 1.07	*w* = 1/[σ^2^(*F*_o_^2^) + (0.0528*P*)^2^ + 0.2372*P*] where *P* = (*F*_o_^2^ + 2*F*_c_^2^)/3
4012 reflections	(Δ/σ)_max_ < 0.001
218 parameters	Δρ_max_ = 0.44 e Å^−^^3^
0 restraints	Δρ_min_ = −0.26 e Å^−^^3^

### Special details

**Table:**

Geometry. All esds (except the esd in the dihedral angle between two l.s. planes) are estimated using the full covariance matrix. The cell esds are taken into account individually in the estimation of esds in distances, angles and torsion angles; correlations between esds in cell parameters are only used when they are defined by crystal symmetry. An approximate (isotropic) treatment of cell esds is used for estimating esds involving l.s. planes.
Refinement. Refinement of F^2^ against ALL reflections. The weighted R-factor wR and goodness of fit S are based on F^2^, conventional R-factors R are based on F, with F set to zero for negative F^2^. The threshold expression of F^2^ > 2sigma(F^2^) is used only for calculating R-factors(gt) etc. and is not relevant to the choice of reflections for refinement. R-factors based on F^2^ are statistically about twice as large as those based on F, and R- factors based on ALL data will be even larger.

### Fractional atomic coordinates and isotropic or equivalent isotropic displacement parameters (Å^2^)

**Table:**

	*x*	*y*	*z*	*U*_iso_*/*U*_eq_	
S1	0.33404 (4)	0.01587 (3)	0.18991 (3)	0.02947 (11)	
O1	0.52084 (12)	0.32470 (10)	0.04883 (10)	0.0334 (2)	
O2	0.45627 (13)	−0.04998 (10)	0.29798 (11)	0.0388 (3)	
C1	0.43524 (16)	0.16549 (13)	0.16271 (14)	0.0279 (3)	
C2	0.55010 (16)	0.26393 (13)	0.26444 (14)	0.0264 (3)	
C3	0.61465 (16)	0.28037 (13)	0.40727 (14)	0.0265 (3)	
H3	0.5837	0.2160	0.4611	0.032*	
C4	0.72549 (16)	0.39299 (13)	0.46990 (14)	0.0273 (3)	
C5	0.76898 (17)	0.48649 (14)	0.38824 (15)	0.0328 (3)	
H5	0.8443	0.5631	0.4321	0.039*	
C6	0.70636 (18)	0.47162 (15)	0.24594 (16)	0.0347 (3)	
H6	0.7366	0.5356	0.1914	0.042*	
C7	0.59801 (17)	0.35912 (14)	0.18809 (14)	0.0291 (3)	
C8	0.42408 (17)	0.20589 (14)	0.03698 (14)	0.0310 (3)	
C9	0.79606 (17)	0.41638 (13)	0.62465 (14)	0.0284 (3)	
H9	0.8821	0.4958	0.6485	0.034*	
C10	0.6659 (2)	0.44576 (18)	0.68066 (16)	0.0416 (4)	
H10A	0.6177	0.5245	0.6381	0.050*	
H10B	0.5769	0.3698	0.6551	0.050*	
C11	0.7379 (2)	0.47091 (17)	0.83653 (16)	0.0423 (4)	
H11A	0.8200	0.5516	0.8617	0.051*	
H11B	0.6496	0.4863	0.8694	0.051*	
C12	0.81830 (19)	0.35505 (15)	0.90597 (15)	0.0368 (3)	
H12A	0.7339	0.2767	0.8892	0.044*	
H12B	0.8697	0.3763	1.0065	0.044*	
C13	0.94782 (19)	0.32296 (16)	0.85200 (15)	0.0364 (3)	
H13A	0.9925	0.2429	0.8941	0.044*	
H13B	1.0391	0.3972	0.8792	0.044*	
C14	0.87798 (18)	0.29943 (14)	0.69594 (14)	0.0315 (3)	
H14A	0.7965	0.2184	0.6695	0.038*	
H14B	0.9674	0.2850	0.6642	0.038*	
C15	0.3295 (2)	0.14926 (17)	−0.10566 (15)	0.0405 (4)	
H15A	0.2767	0.0596	−0.1031	0.061*	
H15B	0.4039	0.1459	−0.1552	0.061*	
H15C	0.2456	0.2049	−0.1528	0.061*	
C16	0.20788 (16)	0.08189 (12)	0.26836 (14)	0.0268 (3)	
C17	0.06675 (19)	0.12795 (16)	0.18436 (16)	0.0387 (3)	
H17	0.0404	0.1284	0.0879	0.046*	
C18	−0.0358 (2)	0.17341 (18)	0.2422 (2)	0.0471 (4)	
H18	−0.1330	0.2058	0.1854	0.057*	
C19	0.0022 (2)	0.17188 (18)	0.3815 (2)	0.0483 (4)	
H19	−0.0685	0.2035	0.4209	0.058*	
C20	0.1428 (2)	0.12449 (18)	0.46414 (17)	0.0462 (4)	
H20	0.1686	0.1236	0.5605	0.055*	
C21	0.24659 (19)	0.07815 (15)	0.40774 (15)	0.0344 (3)	
H21	0.3428	0.0444	0.4644	0.041*	

### Atomic displacement parameters (Å^2^)

**Table:**

	*U*^11^	*U*^22^	*U*^33^	*U*^12^	*U*^13^	*U*^23^
S1	0.0328 (2)	0.02712 (18)	0.02898 (19)	0.00368 (13)	0.01233 (15)	−0.00168 (13)
O1	0.0350 (5)	0.0415 (6)	0.0285 (5)	0.0062 (4)	0.0171 (4)	0.0062 (4)
O2	0.0405 (6)	0.0351 (5)	0.0433 (6)	0.0142 (5)	0.0154 (5)	0.0074 (5)
C1	0.0279 (7)	0.0319 (7)	0.0263 (6)	0.0064 (5)	0.0123 (5)	0.0004 (5)
C2	0.0245 (6)	0.0276 (6)	0.0300 (7)	0.0064 (5)	0.0126 (5)	0.0031 (5)
C3	0.0264 (6)	0.0267 (6)	0.0279 (7)	0.0047 (5)	0.0114 (5)	0.0039 (5)
C4	0.0238 (6)	0.0269 (6)	0.0314 (7)	0.0062 (5)	0.0097 (5)	0.0031 (5)
C5	0.0287 (7)	0.0294 (7)	0.0402 (8)	0.0010 (5)	0.0133 (6)	0.0041 (6)
C6	0.0337 (7)	0.0353 (7)	0.0395 (8)	0.0030 (6)	0.0189 (6)	0.0108 (6)
C7	0.0276 (7)	0.0355 (7)	0.0284 (7)	0.0076 (5)	0.0142 (6)	0.0055 (5)
C8	0.0304 (7)	0.0377 (7)	0.0295 (7)	0.0091 (6)	0.0152 (6)	0.0016 (6)
C9	0.0261 (6)	0.0260 (6)	0.0300 (7)	0.0015 (5)	0.0076 (5)	−0.0001 (5)
C10	0.0355 (8)	0.0534 (9)	0.0348 (8)	0.0191 (7)	0.0080 (7)	−0.0060 (7)
C11	0.0402 (9)	0.0513 (9)	0.0352 (8)	0.0160 (7)	0.0111 (7)	−0.0082 (7)
C12	0.0377 (8)	0.0427 (8)	0.0318 (7)	0.0024 (6)	0.0162 (6)	−0.0022 (6)
C13	0.0371 (8)	0.0437 (8)	0.0305 (7)	0.0133 (7)	0.0127 (6)	0.0059 (6)
C14	0.0343 (7)	0.0335 (7)	0.0301 (7)	0.0099 (6)	0.0143 (6)	0.0040 (5)
C15	0.0449 (9)	0.0518 (9)	0.0279 (7)	0.0110 (7)	0.0159 (7)	−0.0011 (6)
C16	0.0288 (7)	0.0236 (6)	0.0288 (7)	0.0018 (5)	0.0122 (6)	0.0021 (5)
C17	0.0371 (8)	0.0458 (9)	0.0342 (8)	0.0116 (7)	0.0122 (7)	0.0108 (6)
C18	0.0377 (9)	0.0493 (9)	0.0593 (11)	0.0173 (7)	0.0199 (8)	0.0125 (8)
C19	0.0471 (10)	0.0476 (9)	0.0611 (11)	0.0077 (8)	0.0326 (9)	−0.0047 (8)
C20	0.0530 (10)	0.0547 (10)	0.0352 (8)	0.0020 (8)	0.0234 (8)	−0.0054 (7)
C21	0.0338 (7)	0.0393 (8)	0.0282 (7)	0.0028 (6)	0.0099 (6)	0.0009 (6)

### Geometric parameters (Å, °)

**Table:**

S1—O2	1.4843 (11)	C11—H11A	0.9900
S1—C1	1.7529 (14)	C11—H11B	0.9900
S1—C16	1.7955 (14)	C12—C13	1.519 (2)
O1—C8	1.3706 (17)	C12—H12A	0.9900
O1—C7	1.3829 (17)	C12—H12B	0.9900
C1—C8	1.3574 (19)	C13—C14	1.5239 (19)
C1—C2	1.4494 (18)	C13—H13A	0.9900
C2—C7	1.3891 (19)	C13—H13B	0.9900
C2—C3	1.3908 (18)	C14—H14A	0.9900
C3—C4	1.3931 (18)	C14—H14B	0.9900
C3—H3	0.9500	C15—H15A	0.9800
C4—C5	1.4006 (19)	C15—H15B	0.9800
C4—C9	1.5112 (19)	C15—H15C	0.9800
C5—C6	1.385 (2)	C16—C21	1.374 (2)
C5—H5	0.9500	C16—C17	1.380 (2)
C6—C7	1.373 (2)	C17—C18	1.382 (2)
C6—H6	0.9500	C17—H17	0.9500
C8—C15	1.485 (2)	C18—C19	1.372 (3)
C9—C10	1.530 (2)	C18—H18	0.9500
C9—C14	1.5317 (19)	C19—C20	1.379 (3)
C9—H9	1.0000	C19—H19	0.9500
C10—C11	1.524 (2)	C20—C21	1.385 (2)
C10—H10A	0.9900	C20—H20	0.9500
C10—H10B	0.9900	C21—H21	0.9500
C11—C12	1.511 (2)		
			
O2—S1—C1	107.74 (6)	C10—C11—H11B	109.4
O2—S1—C16	106.99 (6)	H11A—C11—H11B	108.0
C1—S1—C16	98.62 (6)	C11—C12—C13	111.07 (13)
C8—O1—C7	106.49 (10)	C11—C12—H12A	109.4
C8—C1—C2	107.37 (12)	C13—C12—H12A	109.4
C8—C1—S1	124.25 (11)	C11—C12—H12B	109.4
C2—C1—S1	128.29 (10)	C13—C12—H12B	109.4
C7—C2—C3	119.49 (12)	H12A—C12—H12B	108.0
C7—C2—C1	104.63 (12)	C12—C13—C14	111.68 (12)
C3—C2—C1	135.87 (12)	C12—C13—H13A	109.3
C2—C3—C4	118.76 (12)	C14—C13—H13A	109.3
C2—C3—H3	120.6	C12—C13—H13B	109.3
C4—C3—H3	120.6	C14—C13—H13B	109.3
C3—C4—C5	119.53 (13)	H13A—C13—H13B	107.9
C3—C4—C9	120.59 (12)	C13—C14—C9	111.88 (12)
C5—C4—C9	119.87 (12)	C13—C14—H14A	109.2
C6—C5—C4	122.53 (13)	C9—C14—H14A	109.2
C6—C5—H5	118.7	C13—C14—H14B	109.2
C4—C5—H5	118.7	C9—C14—H14B	109.2
C7—C6—C5	116.21 (13)	H14A—C14—H14B	107.9
C7—C6—H6	121.9	C8—C15—H15A	109.5
C5—C6—H6	121.9	C8—C15—H15B	109.5
C6—C7—O1	125.85 (12)	H15A—C15—H15B	109.5
C6—C7—C2	123.48 (13)	C8—C15—H15C	109.5
O1—C7—C2	110.67 (12)	H15A—C15—H15C	109.5
C1—C8—O1	110.83 (12)	H15B—C15—H15C	109.5
C1—C8—C15	133.47 (14)	C21—C16—C17	121.28 (13)
O1—C8—C15	115.70 (12)	C21—C16—S1	120.21 (11)
C4—C9—C10	111.77 (11)	C17—C16—S1	118.37 (11)
C4—C9—C14	112.05 (11)	C16—C17—C18	119.21 (15)
C10—C9—C14	109.62 (12)	C16—C17—H17	120.4
C4—C9—H9	107.7	C18—C17—H17	120.4
C10—C9—H9	107.7	C19—C18—C17	120.21 (16)
C14—C9—H9	107.7	C19—C18—H18	119.9
C11—C10—C9	111.62 (13)	C17—C18—H18	119.9
C11—C10—H10A	109.3	C18—C19—C20	120.03 (15)
C9—C10—H10A	109.3	C18—C19—H19	120.0
C11—C10—H10B	109.3	C20—C19—H19	120.0
C9—C10—H10B	109.3	C19—C20—C21	120.49 (16)
H10A—C10—H10B	108.0	C19—C20—H20	119.8
C12—C11—C10	111.23 (13)	C21—C20—H20	119.8
C12—C11—H11A	109.4	C16—C21—C20	118.77 (15)
C10—C11—H11A	109.4	C16—C21—H21	120.6
C12—C11—H11B	109.4	C20—C21—H21	120.6
			
O2—S1—C1—C8	131.22 (12)	C7—O1—C8—C1	1.01 (15)
C16—S1—C1—C8	−117.76 (13)	C7—O1—C8—C15	−179.63 (12)
O2—S1—C1—C2	−45.06 (13)	C3—C4—C9—C10	−68.63 (16)
C16—S1—C1—C2	65.96 (13)	C5—C4—C9—C10	110.04 (15)
C8—C1—C2—C7	0.77 (15)	C3—C4—C9—C14	54.86 (16)
S1—C1—C2—C7	177.55 (11)	C5—C4—C9—C14	−126.47 (14)
C8—C1—C2—C3	−179.30 (15)	C4—C9—C10—C11	−179.32 (13)
S1—C1—C2—C3	−2.5 (2)	C14—C9—C10—C11	55.83 (17)
C7—C2—C3—C4	0.36 (19)	C9—C10—C11—C12	−56.99 (19)
C1—C2—C3—C4	−179.57 (14)	C10—C11—C12—C13	55.67 (18)
C2—C3—C4—C5	0.07 (19)	C11—C12—C13—C14	−54.82 (17)
C2—C3—C4—C9	178.73 (11)	C12—C13—C14—C9	55.03 (17)
C3—C4—C5—C6	−0.3 (2)	C4—C9—C14—C13	−179.56 (12)
C9—C4—C5—C6	−178.94 (13)	C10—C9—C14—C13	−54.87 (16)
C4—C5—C6—C7	0.0 (2)	O2—S1—C16—C21	6.99 (13)
C5—C6—C7—O1	179.83 (12)	C1—S1—C16—C21	−104.63 (12)
C5—C6—C7—C2	0.4 (2)	O2—S1—C16—C17	−168.86 (11)
C8—O1—C7—C6	−179.94 (14)	C1—S1—C16—C17	79.52 (12)
C8—O1—C7—C2	−0.49 (15)	C21—C16—C17—C18	1.2 (2)
C3—C2—C7—C6	−0.6 (2)	S1—C16—C17—C18	177.02 (13)
C1—C2—C7—C6	179.30 (13)	C16—C17—C18—C19	−0.4 (3)
C3—C2—C7—O1	179.89 (11)	C17—C18—C19—C20	−0.2 (3)
C1—C2—C7—O1	−0.16 (14)	C18—C19—C20—C21	0.0 (3)
C2—C1—C8—O1	−1.12 (15)	C17—C16—C21—C20	−1.4 (2)
S1—C1—C8—O1	−178.06 (9)	S1—C16—C21—C20	−177.15 (12)
C2—C1—C8—C15	179.67 (15)	C19—C20—C21—C16	0.8 (2)
S1—C1—C8—C15	2.7 (2)		

### Hydrogen-bond geometry (Å, °)

**Table:**

*D*—H···*A*	*D*—H	H···*A*	*D*···*A*	*D*—H···*A*
C15—H15B···O2^i^	0.98	2.52	3.4516 (19)	159
C21—H21···O2^ii^	0.95	2.47	3.2793 (18)	144

Symmetry codes: (i) −*x*+1, −*y*, −*z*; (ii) −*x*+1, −*y*, −*z*+1.
